# Exploring the Impact of Online Mental Health Resources During the COVID-19 Pandemic on Lesbian, Gay, Bisexual, Transgender, Queer, and Questioning Adults Compared to Heterosexual Adults: Pretest-Posttest Survey Analyses

**DOI:** 10.2196/67082

**Published:** 2025-07-11

**Authors:** Natalia Ramos, Skylar Jones, Lily Zhang, Miriam Nuño, Benita Ramsey, Dannie Ceseña, Alyssa Mireles, Kenneth Wells

**Affiliations:** 1 Department of Psychiatry and Biobehavioral Sciences David Geffen School of Medicine University of California, Los Angeles Los Angeles, CA United States; 2 Research Center for Health Services and Society Jane and Terry Semel Institute for Neuroscience and Human Behavior University of California, Los Angeles Los Angeles, CA United States; 3 Department of Public Health Sciences University of California Davis Davis, CA United States; 4 Rainbow Pride Youth Alliance San Bernadino, CA United States; 5 California LGBTQ Health Human Services Network Carson, CA United States; 6 Center for Health Services and Society Jane and Terry Semel Institute for Neuroscience and Human Behavior University of California, Los Angeles Los Angeles, CA United States; 7 Department of Health Policy and Management Fielding School of Public Health University of California, Los Angeles Los Angeles, CA United States

**Keywords:** digital mental health, depression, anxiety, prevention, COVID-19, lesbian, gay, bisexual, transgender, queer, and questioning, LGBTQ+, well-being resources

## Abstract

**Background:**

Lesbian, gay, bisexual, transgender, queer, and questioning (LGBTQ+) individuals faced greater mental health challenges during the COVID-19 pandemic than binary-gender heterosexual (non-LGBTQ+) adults. The Together for Wellness/Juntos por Nuestro Bienestar website with free well-being resources, developed during the COVID-19 pandemic with partner input, included LGBTQ+ resources. A pilot evaluation among adults (aged ≥18 years) found engagement with and use of the website 4 to 6 weeks before follow-up was associated with reduced (pretest-posttest) depression. Results for LGBTQ+ participants were not reported.

**Objective:**

This study describes baseline depression, anxiety, and website engagement for LGBTQ+ compared with non-LGBTQ+ adults and pretest-posttest changes in depression and anxiety (the primary outcome).

**Methods:**

Community partners invited health and social services providers, clients, and partners to visit the website and complete a survey app (Chorus Innovations) at baseline (September 20, 2021-April 4, 2022) and a 4- to 6-week follow-up (October 22, 2021-May 17, 2022). LGBTQ+ adults were compared to non-LGBTQ+ adults in demographics, website use, depression, and anxiety. Sensitivity analyses were adjusted for nonresponse (inverse probability weighting). Regression analyses identified predictors for reduction (pretest-posttest) in depression (2-item Patient Health Questionnaire [PHQ-2]) and anxiety (2-item Generalized Anxiety Disorder scale [GAD-2]).

**Results:**

Of 315 adults who completed the baseline survey and 193 who completed the follow-up survey, 64 (20.3%) and 37 (19.2%), respectively, were LGBTQ+. At baseline, LGBTQ+ compared to non-LGBTQ+ adults had higher scores on the PHQ-2 (mean 2.4, SD 1.7 vs 1.3, SD 1.3; *t*_294_=5.31; *P*<.001) and GAD-2 (mean 2.7, SD 1.7 vs 1.6, SD 1.5; *t*_295_=4.96; *P*<.001) and more COVID-19 stressors (mean score 8.1, SD 4.4 vs 6.5, SD 4.0; *t*_298_=2.8; *P*=.003). Before follow-up, LGBTQ+ adults had similar website use (*P*=.65) and likelihood to recommend the website to others (*P*=.26) compared to non-LGBTQ+ adults. LGBTQ+ adults had more reduction (pretest-posttest) in mean GAD-2 scores (−0.8, SD 2.0 vs 0.0, SD 1.2; *t*_177_=−3.08; *P*=.002) and mean PHQ-2 scores (−0.7, SD 1.7 vs −0.1, SD 1.4; *t*_180_=−2.16; *P*=.03) compared to non-LGBTQ+ adults. For LGBTQ+ adults, predictors of pretest-posttest decline (adjusting for nonresponse) in mean GAD-2 scores included visiting the website and using resources 4 to 6 weeks before (β=−1.95, 95% CI −3.20 to −0.70; *P*=.003); for decline in mean PHQ-2, visiting website/using resources had a trend as predictor that was not significant adjusting for nonresponse (β=-.94 (-2.00, 0.013), *P*=.09).

**Conclusions:**

LGBTQ+ adults reported higher baseline depression, anxiety, and COVID-19 stressors than non-LGBTQ+ adults. Among LGBTQ+ but not among non-LGBTQ+ adults, higher website use was associated with reduced anxiety over time. Findings suggest that online resources may promote well-being for LGBTQ+ adults in pandemics.

## Introduction

### Background

Lesbian, gay, bisexual, transgender, queer, and questioning (LGBTQ+) individuals experience significant inequities in mental health indicators. Research studies consistently demonstrate high rates of depression, anxiety, suicidal thoughts and behaviors, and nonsuicidal self-injury among LGBTQ+ adolescents and adults [1-3]. US data for the 7 years before the COVID-19 pandemic showed that LGBTQ+ adults aged 18 to 65 years had higher odds of alcohol and drug use disorder than their cisgender heterosexual peers [4]. Minority stress theory explains that these mental health inequities result from experiences of identity-linked stigma and discrimination—for example, employment discrimination, anti-LGBTQ+ legislation [5], and prejudiced statements—across social settings and over sustained periods [6,7]. Furthermore, LGBTQ+ individuals face unique barriers in mental health care settings, including identity disclosure [8,9]; low rates of health insurance coverage [10] difficulty finding well-trained, gender-affirming mental health care providers [11].

During the COVID-19 pandemic, US and international studies alike showed greater increases in stress, anxiety, and depression among LGBTQ+ individuals as compared to their non-LGBTQ+ peers, with variations observed among specific LGBTQ+ and gender-diverse subpopulations [12-17]. LGBTQ+ young adults in the United States had a disproportionate prevalence of major depressive disorder and generalized anxiety disorder [18]. These amplified mental health challenges faced by LGBTQ+ communities may be attributable to (1) the disproportionate burden of social stressors impacting already less resourced and more marginalized communities and (2) reduced access to social and community support networks [19] and health care resources known to buffer stressors among LGBTQ+ populations. Both general social support and identity-specific support help prevent anxiety and depression among gender-diverse individuals [20-22]. Unsurprisingly, data suggest that social isolation may have contributed to anxiety, stress, and fear during the pandemic [23].

Pandemic data reveal disproportionate mental health impacts across different gender identities and sexual orientations. During the pandemic, women reported experiencing more stress and a higher need for mental health support compared to men [24-26]. Gender-diverse individuals experienced more severe depression, anxiety, and stress than cisgender respondents in a cross-sectional Canadian study conducted early in the pandemic [27]. LGBTQ+ adults experienced greater psychological distress and peritraumatic stress than cisgender heterosexual adults [16]. Although most communities experienced lowered access to mental health care during pandemic shutdowns, LGBTQ+ individuals may have been especially impacted given the compounded barriers to care and a higher baseline need. Gender-diverse (eg, transgender, nonbinary, and genderqueer) individuals also faced unique stress related to poor access to gender-affirming care [27]. In addition, the COVID-19 pandemic may have disproportionately affected younger populations, which have a higher representation of LGBTQ+ individuals [19].

### Digital Resources

Digital tools may serve as an important resource to support the well-being of LGBTQ+ individuals, who often face pronounced social stressors, isolation from supportive spaces, and limited care access both during periods of reduced socialization such as the COVID-19 pandemic and in their everyday lives. The demand for digital mental health services increased substantially worldwide during the pandemic given the strain on already overburdened mental health systems [28-30]. Digital resources providing health care services and social support took on many forms during the pandemic, including videoconferencing, educational websites, mobile health apps, SMS text messaging, and online chats (live or chatbots) [31]. Most digital technologies were web based, although supportive mobile SMS text messaging and online chats were also offered in multiple countries [19]. In Australia, efforts to integrate digital youth portals focused on mental illness prevention launched via existing brick-and-mortar youth mental health centers in an effort to expand access across rural areas and decrease barriers to care [20]. A systematic review of LGBTQ+-specific digital resources for youth during the pandemic found that both structured and unstructured formal resources—such as telehealth and mobile apps—reduced mental illness symptoms despite needed improvement of mobile apps [22]. In addition, remote services may enhance access to LGBTQ+-affirming substance use treatment and harm reduction services [32].

Despite the growth in interest and demand for digital mental health resources, data on the effectiveness of digital interventions for mental health remain limited [30]. A 2016 systematic review of depression apps found that only 10% integrated evidence-based interventions such as cognitive behavioral therapy and behavioral activation [33]. Digital resources, of increased importance during the COVID-19 pandemic [34], often face limitations in their ability to be tailored to specific communities and cultural contexts [34,35]. To maximize the effectiveness of digital interventions, it is crucial to consider population-specific factors and the unique needs of users [34-36]. For LGBTQ+ individuals experiencing depression and anxiety symptoms, digital interventions can help address the emotional and behavioral impacts of stigma-related events [36]. Community-partnered development of digital resources has previously been proposed as a potentially effective model for creating accessible, culturally relevant mental health content that fosters strong community engagement and uptake [37].

### The Together for Wellness/Juntos por Nuestro Bienestar Initiative

Together for Wellness/Juntos por Nuestro Bienestar (T4W/Juntos) is a free public website focused on mental health and well-being during the COVID-19 pandemic. Funded by the California Health Care Services Division of Behavioral Health, T4W/Juntos was developed through an academic-community partnership that focused on meeting the needs of marginalized and underresourced communities affected disproportionately by COVID-19, including LGBTQ+ communities. The site offered resources that were evidence informed or evidence based, developed in collaboration with diverse partner agencies and reviewed following the principles of community-partnered participatory research [38]. The details of the initiative’s community-partnered approach and conceptual framework combining the technology acceptance model and vulnerable populations framework for engagement in healthy equity–focused prevention resources are described elsewhere [39-43]. The website offered resources on stress, grief, social connections, racial discrimination, COVID-19, and support for families, teachers, and older adults during this evaluation phase [42]. A total of 133 resources were offered across various digital formats, including websites, videos, YouTube links, PDF files, apps, and hotlines, with some content available in 13 languages.

A pilot evaluation recruited adults through community partner email invitations. Participants were invited to consent, engage with a research duplicate version of the public website, and fill out surveys at baseline and after 4 to 6 weeks. Previous analyses on the sample of all respondents found significant levels of COVID-19 stressors, depression, and anxiety for adults (both English and Spanish speaking), and greater use of the website was associated with a reduction in depression for the overall sample [43]. However, the previous analyses and reports did not present findings on LGBTQ+ participants separately or in relation to non-LGBTQ+ peers. The goals of this analysis were to describe LGBTQ+ participants in the T4W/Juntos pilot evaluation and compare their use of the website and depression (2-item Patient Health Questionnaire [PHQ-2]) and anxiety (2-item Generalized Anxiety Disorder scale [GAD-2]) levels to those of non-LGBTQ+ participants [44,45]. We hypothesized that, at baseline, LGBTQ+ participants would report higher levels of COVID-19–related stress, depression, and anxiety compared to non-LGBTQ+ respondents. We further hypothesized that LGBTQ+ participants would exhibit decreased depression and anxiety symptoms with greater use of the website before follow-up (as in the main analysis for the overall sample for depression). Other significant predictors of pretest-posttest decline in depression and anxiety were also explored.

### Measuring Sexual Orientation and Gender Identity

Previous research on surveying LGBTQ+ identities has emphasized the importance of affirming language, recognizing the fluidity of identity over time, assessing comfort with disclosure, and highlighting the role of community support and gender-affirming services [46,47]. While previous studies have inconsistently captured identity-related factors, some literature suggests assessing gender and sexual orientation across 3 dimensions: identity, behavior, and attraction [47]. Few studies adhere to the 2022 National Institutes of Health (NIH) recommendations to capture at least 2 of the 3 key domains [48]. For gender identity, many federally funded initiatives use the NIH-recommended 2-step process, using separate questions to survey both *sex assigned at birth* and current *gender identity* [48]. However, the category of *sex assigned at birth* has its own limitations given that it is determined solely by the appearance of the infant’s external genitalia at birth, overlooking biological variations in sex traits such as chromosomes, sex hormones, and internal anatomy [49]. Furthermore, some transgender or intersex individuals and community partners also find the term problematic [50,51].

## Methods

### T4W/Juntos Website Resources

As explained previously and in a previous publication, the website was developed with partner input from 11 agencies, including 2 serving LGBTQ+ populations, with feedback from 4 focus groups providing input on final design [42]. The resources were largely evidence informed, including mindfulness apps, as there are few randomized trials of online resources (eg, websites and apps) designed for specific populations or tested broadly across multiple population [52-55].

### Evaluation Design

The website evaluation was conducted by having 11 California community-based agencies who signed participation agreements email invites to a prespecified number of participants (clients, community partners, and health and social services providers) on their existing lists. For those with large email lists, a random selection tool was provided. The number of invites was initially 80 to 100 per agency to enroll 30 to 40 participants, later expanded to 70 to have 300 to 350 eligible participants. Participants followed a link (through a Chorus Innovations app) to review an information sheet and consent form, and those who agreed were sent a link to complete a baseline survey and review a research duplicate of the public website only for invited participants and then invited to complete a 4- to 6-week follow-up survey. The research duplicate website was also used by the inviting agencies to track enrollment.

The survey period was September 20, 2021, to April 4, 2022, for baseline and October 22, 2021, to May 17, 2022, for follow-up.

### Ethical Considerations

This study was reviewed and approved by the University of California, Los Angeles Institutional Review Board for human participants (20-002163-AM-00008). All participants provided informed consent online and were advised that participation was voluntary. Data were deidentified, with contact information for follow-up and linking data stored on a secure server separately from study data. Informed consent was affirmed at follow-ups. For each event, participants received a US $25 e-gift card. The inviting agencies were not informed of the individuals participating.

### Measures

#### Baseline

Demographics included age in years, race or ethnicity (American Indian, Native American, or Alaskan; Black, African American, or African; East Asian; South East Asian; South Asian; Hispanic, Latino, or Spanish origin; Middle Eastern; Pacific Islander; White, Caucasian, or European; unknown; or not stated), website language preference (English, Spanish, Cantonese, Vietnamese, Tagalog, Mandarin, Korean, Japanese, Russian, Farsi, Armenian, Arabic, Mixteco, other, unknown, or not stated), educational level (some high school or lower, high school graduate or equivalent, vocational education or certificate, some college, college graduate, or graduate school), and zip code [56-59]. In total, 2 mental health stigma items were included at baseline (5-point Likert agreement scale plus “don’t know”) [56].

In consultation with community partners, we selected two items to capture gender and sexual identities: (1) gender identity (female, male, genderqueer, questioning, trans man, trans woman, other, unknown, and prefer not to state) and (2) sexual orientation (straight, gay/lesbian, bisexual/pansexual, queer, not sexual/none, questioning, other, unknown, and prefer not to state). For our analysis, we categorized participants as LGBTQ+ if they identified with a nonbinary gender (female or male) or a nonheterosexual orientation. For analyses, we excluded individuals with missing data, including those who selected “prefer not to state” or “unknown,” to explicitly focus on those directly describing their gender and sexual identities. For the main analyses, we compared the LGBTQ+ participants to the non-LGBTQ+ participants, and for the sensitivity analyses (Multimedia Appendix 1), we compared 3 groups (LGBTQ+, female heterosexual, and male heterosexual participants) overall and in 2-way analyses. These analyses were designed to inform future studies comparing mental health and stressors across gender groups given evidence that gender identity can influence these outcomes [13,18,23,26,58,59].

#### Baseline and Follow-Up

Depression and anxiety were assessed using mean PHQ-2 and GAD-2 scores (range 0-6; score of ≥3 indicating moderate or greater [high] levels vs a score of <3 [low]), and for the sensitivity analyses as well as fitting predictors into main analyses, a PHQ-2 or GAD-2 score of ≥3 was also included as a predictor in regression analyses if it was more significant than either PHQ-2 or GAD-2 alone [44,45]. Behavioral health service use—including behavioral health hotline use and any behavioral health service use (emergency room, primary care, mental health care, substance use visits, hospitalization, and residential care)—was assessed for the previous 6 months at baseline and for the previous month at follow-up [56]. Pandemic-related stress at baseline was measured based on the overall impact of COVID-19 as assessed using 5 categories. We used an adapted version of the COVID-19 Stress Scale to capture related behavior changes (social distancing, isolation, caring for someone at home, working from home, not working, change in health care services, following media coverage on COVID-19, and changing travel plans) [60]. We used an adapted version of COVID-19 stressors [58] that captured having COVID-19; fear of acquiring or spreading COVID-19; worrying about others; stigma or discrimination; financial, food, and home insecurity; frustration, depression, anxiety, alcohol use, sleep, and sexual activity change; confusion about COVID-19; and other difficulties (not contributing to the greater good, having social and emotional support, and having financial support from others) [58,60,61]. We included an additional measure of racial and ethnic discrimination during the COVID-19 pandemic (6 items; 4-point Likert scale [62]). For COVID-19–related stressors, we used the total score and explored the individual stressors noted previously. We also included date of survey completion.

Website use and engagement at baseline were assessed using website satisfaction, relevance, and ease of use (mean score of 3 items), as well as comfort using the website (separate item). Each item used a 5-point Likert agreement scale plus a “don’t know” option. At follow-up, website use and engagement were assessed based on (1) any use in the previous month (yes or no); (2) a total score of use summed across all 6 categories of resources, each with 5 response options (“did not use,” “used some,” “used and valuable,” “used and very valuable,” and “used and would recommend to anyone”); and (3) whether they would recommend it to others (and, if so, the number of times they recommended it and which resource categories were recommended) [37,43,56].

### Statistical Analyses

We conducted analyses for the LGBTQ+ participants compared to non-LGBTQ+ participants (those identifying as having a male or female gender and heterosexual) as main analyses. For sensitivity analyses (Multimedia Appendix 1), we included a 3-way comparison of LGBTQ+, female heterosexual, and male heterosexual groups with 2-way comparisons within the 3 groups (Tables S1-S4 in Multimedia Appendix 1). Chi-square tests were used for categorical variables, and *t* tests (2-tailed) were used for continuous variables to compare the 2 groups. To describe the samples, we used means for continuous variables and counts and percentages for categorical variables. For preliminary bivariate analyses, the predictors tested included demographics, depression and anxiety, mental health stigma, COVID-19–related behaviors and stressors, and behavioral health service use (including adjustment for follow-up nonresponse). The final regression models included predictors significant at *P*<.05.

For website use in the month before follow-up, we used logistic regression for any use and linear regression for the total score of use across the 6 categories. We considered significant predictors of pretest-posttest difference in PHQ-2 and GAD-2 mean scores as our main analysis. We initially examined all measures as predictors in bivariate analyses, including predictors of follow-up nonresponse. We fit regression models using significant individual predictors, and when including predictors, retaining only those that remained significant through sequential testing to inform the final models. Regarding the final follow-up models on website use and mental health, for the sensitivity analyses, we applied inverse probability weighting to adjust for nonresponse predictors at baseline (age) and follow-up [63,64]. All main analyses were conducted for LGBTQ+ versus non-LGBTQ+ participants. As a sensitivity analysis, we conducted 3-way overall and separate analyses for LGBTQ+, female heterosexual, and male heterosexual groups, with results and tables provided in Multimedia Appendix 1. Statistical results appear in tables (and Multimedia Appendix 1) and in the text when cited or not tabled.

## Results

### Sample

Of the 315 respondents at baseline, 236 (74.9%) identified as heterosexual, 17 (5.4%) identified as gay or lesbian, 31 (9.8%) identified as bisexual or pansexual, 8 (2.5%) identified as queer, 2 (0.6%) selected the option “not sexual/none,” 2 (0.6%) identified as questioning, and 4 (1.3%) identified as “other.” Regarding gender identity, of the 315 respondents, 225 (71.4%) identified as female, 70 (22.2%) identified as male, and 15 (4.8%) identified as gender diverse (including genderqueer, questioning, trans man, trans woman, and “other”). In total, 4.8% (15/315) of the participants skipped the sexual orientation or gender identity items or selected “don’t know” or “prefer not to say”; these individuals were not included in the analyses, for a total sample for analysis of 300.

### Baseline Characteristics

As shown in Table 1, LGBTQ+ participants were significantly younger than non-LGBTQ+ participants (mean age 33.1, SD 12.4 years vs 40.4, SD 13.6 years; t_298_=−3.88; *P*<.001). Website language preference differed (χ^2^_2_=6.3; *P*=.04), with LGBTQ+ participants having a higher preference for English (57/73, 78% vs 191/235, 81.3%) and a lower preference for Spanish (4/63, 6% vs 32/235, 13.6%) or other language (0% vs 12/235, 5.1%) than non-LGBTQ+ participants. LGBTQ+ and non-LGBTQ+ participants did not differ significantly in race or ethnicity (χ^2^_3_=3.2; *P*=.36) and educational level (χ^2^_4_=0.8; *P*=.93). LGBTQ+ participants had significantly higher rates of baseline depression (mean PHQ-2 score 2.4, SD 1.7 vs 1.3, SD 1.3; t_294_=5.31; *P*<.001) and anxiety (mean GAD-2 score 2.7, SD 1.7 vs 1.6, SD 1.5; t_295_=4.96; *P*<.001) than non-LGBTQ+ participants. LGBTQ+ participants had a higher use of behavioral health services than non-LGBTQ+ participants (33/41, 80% vs 82/146, 56.2%; χ^2^_1_=8.0; *P*=.005; imputing for missing data: 33/48, 69% vs 82/181, 45.3%; *P*=.004) and a significantly higher number of COVID-19 stressors (mean score 8.1, SD 4.4 vs mean 6.5, SD 4.0; t_298_=2.8; *P*=.005). There were no significant differences in mean website engagement (3 items; mean score 4.0, SD 0.7 vs 4.1, SD 0.7; t_288_=−1.02; *P*=.31), comfort with website use (χ^2^_4_=3.3; *P*=.51), or mean mental health stigma (mean score 3.0, SD 0.9 vs 2.8, SD 1.0; t_275_=1.48; *P*=.14).

**Table 1:** Characteristics (baseline survey; September 2021-April 2022) of the California Together for Wellness/Juntos por Nuestro Bienestar website study participants—lesbian, gay, bisexual, transgender, queer, and questioning (LGBTQ+)^a^ versus non-LGBTQ+^b^ participants (N=300).

Baseline characteristic		Overall	Male or female heterosexual participants (n=236)	LGBTQ+ participants (n=64)	Statistic^c^		*P* value
					*t* test (*df*)	Chi-square (*df*)	
Age (y), mean (SD)		38.9 (13.7)	40.4 (13.6)	33.1 (12.4)	−3.88 (298)	—^d^	<.001
**Gender, n (%)**					—	59.5 (2)	<.001
	Female	217 (72.3)	183 (77.5)	34 (53.1)			
	Male	68 (22.7)	53 (22.5)	15 (23.4)			
	Other	15 (5)	0 (0)	15 (23.4)			
**Race, n (%)**					—	3.2 (3)	.36
	Black or African American	53 (18.4)^e^	40 (17.6)^f^	13 (21.3)^g^			
	Hispanic, Latinx, or Spanish origin	125 (43.4)^e^	104 (45.8)^f^	21 (34.4)^g^			
	White	79 (27.4)^e^	58 (25.6)^f^	21 (34.4)^g^			
	Other	31 (10.8)^e^	25 (11)^f^	6 (9.8)^g^			
**Educational level, n (%)**					—	0.8 (4)	.93
	Lower than high school	21 (7)^h^	16 (6.8)^i^	5 (7.9)^j^			
	High school graduate	34 (11.4)^h^	28 (11.9)^i^	6 (9.5)^j^			
	Some college	84 (28.2)^h^	68 (28.9)^i^	16 (25.4)^j^			
	College	113 (37.9)^h^	88 (37.4)^i^	25 (39.7)^j^			
	Graduate school	46 (15.4)^h^	35 (14.9)^i^	11 (17.5)^j^			
**Language preferred for website use, n (%)**					—	6.3 (2)	.04
	English	250 (83.9)^h^	191 (81.3)^i^	59 (93.7)^j^			
	Spanish	36 (12.1)^h^	32 (13.6)^i^	4 (6.3)^j^			
	Other	12 (4)^h^	12 (5.1)^i^	0 (0)^j^			
PHQ-2^k^ score, mean (SD)		1.6 (1.4)	1.3 (1.3)	2.4 (1.7)	5.31 (294)	—	<.001
**PHQ-2 score, n (%)**					—	22.4 (1)	<.001
	High (≥3)	57 (19.3)^l^	32 (13.7)^m^	25 (40.3)^n^			
	Low	239 (80.7)^l^	202 (86.3)^m^	37 (59.7)^n^			
GAD-2^o^ score, mean (SD)		1.8 (1.6)	1.6 (1.5)	2.7 (1.7)	4.96 (295)	—	<.001
**GAD-2 score, n (%)**					—	23.4 (1)	<.001
	High (≥3)	76 (25.6)^p^	45 (19.2)^m^	31 (49.2)^j^			
	Low	221 (74.4)^p^	189 (80.8)^m^	32 (50.8)^j^			
Stigma score, mean (SD)		2.8 (1.0)	2.8 (1.0)	3.0 (0.9)	1.48 (275)	—	.14
Engagement score (3 items)^q^, mean (SD)		4.0 (0.7)	4.1 (0.7)	4.0 (0.7)	−1.02 (288)	—	.31
**Did not feel comfortable using the website, n (%)**					—	3.3 (4)	.51
	Strongly disagree	99 (34)^r^	74 (32.3)^s^	25 (40.3)^n^			
	Disagree	105 (36.1)^r^	81 (35.4)^s^	24 (38.7)^n^			
	Neither agree nor disagree	22 (7.6)^r^	19 (8.3)^s^	3 (4.8)^n^			
	Agree	36 (12.4)^r^	30 (13.1)^s^	6 (9.7)^n^			
	Strongly agree	29 (10)^r^	25 (10.9)^s^	4 (6.5)^n^			
**Any service use for emotional, mental health, alcohol, or drug problems, n (%)**					—	8 (1)	.005
	Yes	115 (61.5)^t^	82 (56.2)^u^	33 (80.5)^v^			
	No	72 (38.5)^t^	64 (43.8)^u^	8 (19.5)^v^			
**Any service use for emotional, mental health, alcohol, or drug problems (imputed)^w^, n (%)**					—	8.3 (1)	.004
	Yes	115 (50.2)^s^	82 (45.3)^x^	33 (68.8)^y^			
	No	114 (49.8)^s^	99 (54.7)^x^	15 (31.3)^y^			
Total number of COVID-19–related behavior changes, mean (SD)		3.9 (2.0)	3.8 (1.9)	4.0 (2.2)	0.88 (298)	—	.38
Total number of COVID-19 stressors experienced, mean (SD)		6.9 (4.1)	6.5 (4.0)	8.1 (4.4)	2.8 (298)	—	.005

^a^This category captures those with a gender identity other than male or female or a sexual orientation other than heterosexual (excluding missing values or “don’t know” or “prefer not to state” response options).

^b^The non-LGBTQ+ category includes participants who identified as heterosexual in their sexual orientation *and* with a binary gender identity (either male or female). None of these participants disclosed a transgender or gender-diverse identity.

^c^Chi-square tests were used for categorical variables, and *t* tests were used for continuous variables to compare the 2 groups with and without follow-up response.

^d^Not applicable.

^e^n=288.

^f^n=227.

^g^n=61.

^h^n=298.

^i^n=235.

^j^n=63.

^k^PHQ-2: 2-item Patient Health Questionnaire.

^l^n=296.

^m^n=234.

^n^n=62.

^o^GAD-2: 2-item Generalized Anxiety Disorder scale.

^p^n=297.

^q^Items (ease of use, relevance of topics, and satisfaction) were averaged as mean engagement based on 5-point Likert scales.

^r^n=291.

^s^n=229.

^t^n=187.

^u^n=146.

^v^n=41.

^w^The inpatient or rehabilitation item in this set was assigned 0 if it was skipped but the item on other use was answered. The imputed version was for the sensitivity analysis.

^x^n=181.

^y^n=48.

### Baseline to Follow-Up Response

The mean number of days between baseline and follow-up for the sample that completed both (185/300, 61.7%) was 34.6 (SD 10.8) and was a borderline predictor, although it was not statistically significant, for longer follow-up for LGBTQ+ participants (37/185, 20%; mean 37.5, SD 13.2 days) versus non-LGBTQ+ participants (148/185, 80%; mean 33.9, SD 10.2 days; t_183_=−1.81; *P*=.07). For follow-up completion (October 2021-May 2022) by baseline sexual orientation and gender identity for the California T4W/Juntos study participants (N=300): for the LGBTQ+ sample (n=64, 23% total sample), there were 27 (42%) with no follow-up and 37 (58%) with follow-up data (χ^2^_1_=0.5; *P*=.48). For Non-LGBTQ+ (overall n=236, 78.7% total sample) there were 88 (37.3%) with no follow-up data and 148 (62.7%) for having follow-up data (χ^2^_1_=0.5, *P*=.48). The difference in follow-up response between LGBTQ+ and non-LGBTQ+ participants was not significant (27/64, 42% vs 88/236, 37.3%, respectively; χ^2^_1_=0.5; *P*=.48). Among LGBTQ+ participants, at least one respondent in the follow-up sample selected each gender identity and sexual orientation response at baseline.

### Follow-Up Descriptive Characteristics

At follow-up (Table 2), there was no significant difference between LGBTQ+ and non-LGBTQ+ participants in the percentage of those who visited the website or used the resources in the preceding 4 to 6 weeks (24/37, 65% vs 90/148, 60.8%; χ^2^_1_=0.2; *P*=.65), the total score of the categories viewed or used (mean 12.9, SD 7.7 vs mean 11.6, SD 6.2; t_176_=1.1; *P*=.27), or the percentage of those who recommended the website to others (19/27, 70% vs 91/148, 61.5%; χ^2^_1_=1.26; *P*=.26). The total COVID-19 stressor score at follow-up was greater for LGBTQ+ participants than for non-LGBTQ+ participants (mean 8.5, SD 4.4 vs mean 6.6, SD 4.2; t_183_=2.42; *P*=.02). For 2 weeks before follow-up, LGBTQ+ participants did not differ significantly from non-LGBTQ+ participants in depression (mean PHQ-2 score 1.8, SD 1.6 vs 1.4, SD 1.3; t_183_=1.77; *P*=.08) or anxiety (mean GAD-2 score 2.1, SD 2.0 vs 1.6, SD 1.5; t_180_=1.41; *P*=.16).

**Table 2:** Characteristics of the California Together for Wellness/Juntos por Nuestro Bienestar (T4W/Juntos) study participants—lesbian, gay, bisexual, transgender, queer, and questioning (LGBTQ+)^a^ versus non-LGBTQ+^b^ at survey follow-up (October 2021-May 2022; N=185).

Characteristic (follow-up)			Overall	Non–gender diverse and heterosexual participants (n=148)	LGBTQ+ participants (n=37)	Statistic^c^		*P* value
						*t* test (*df*)	Chi-square (*df*)	
**Visited the** T4W/Juntos **site or used the resources 4-6 wk before, n (%)**						—^d^	0.2 (11)	.65
	Yes		114 (61.6)	90 (60.8)	24 (64.9)			
	No		71 (38.4)	58 (39.2)	13 (35.1)			
Total score of T4W/Juntos resource categories viewed or used 4-6 wk before, mean (SD)			11.9 (6.5)	11.6 (6.2)	12.9 (7.7)	1.1 (176)	—	.27
**Recommended the website to others (4-6 wk before), n (%)**						—	1.3 (1)	.26
	Yes		110 (59.5)	91 (61.5)	19 (51.4)			
	No		75 (40.5)	57 (38.5)	18 (48.6)			
Number of times recommended^e^, mean (SD)			3.0 (4.6)	3.0 (4.4)	3.1 (5.3)	−0.09	—	.93
**Follow-up mental health variables**								
	PHQ-2^f^ score, mean (SD)		1.5 (1.4)	1.4 (1.3)	1.8 (1.6)	1.77 (183)	—	.08
	**PHQ-2 score, n (%)**					—	6.0 (1)	.01
		High (≥3)	38 (20.5)	25 (16.9)	13 (35.1)			
		Low	147 (79.5)	123 (83.1)	24 (64.9)			
	PHQ-2 pretest-posttest score change, mean (SD)		−0.2 (1.5)	−0.1 (1.4)	−0.7 (1.7)	−2.16 (180)	—	.03
	GAD-2^g^ score, mean (SD)		1.7 (1.6)	1.6 (1.5)	2.1 (2.0)	1.41 (180)	—	.16
	**GAD-2 score, n (%)**					—	2.2 (1)	.14
		High (≥3)	39 (21.4)^h^	28 (19.2)^i^	11 (30.6)^j^			
		Low	143 (78.6)^h^	118 (80.8)^i^	25 (69.4)^j^			
	GAD-2 pretest-posttest score change, mean (SD)		−0.2 (1.4)	0.0 (1.2)	−0.8 (2.0)	−3.08 (177)	—	.002
	Stigma score, mean (SD)		2.9 (1.0)	2.9 (1.0)	2.8 (1.1)	−0.88 (177)	—	.38
	**Any service use for emotional, mental health, alcohol, or drug problems, n (%)**					—	5.2 (1)	.02
		Yes	53 (43.8)^k^	37 (38.5)^l^	16 (64)^m^			
		No	68 (56.2)^k^	59 (61.5)^l^	9 (36)^m^			
	**Any service use for emotional, mental health, alcohol, or drug problems (imputed)^n^, n (%)**					—	5.7 (1)	.02
		Yes	53 (36.1)^o^	37 (31.4)^p^	16 (55.2)^q^			
		No	94 (63.9)^o^	81 (68.6)^p^	13 (44.8)^q^			
	Total number of COVID-19 stressors experienced, mean (SD)		7.0 (4.3)	6.6 (4.2)	8.5 (4.4)	2.42 (183)	—	.02

^a^This category captures those with a gender identity other than male or female or a sexual orientation other than heterosexual (excluding missing values or “don’t know” or “prefer not to state” response options).

^b^The non-LGBTQ+ category includes participants who identified as heterosexual in their sexual orientation *and* with a binary gender identity (either male or female). None of these participants disclosed a transgender or gender-diverse identity.

^c^Chi-square tests were used for categorical variables, and *t* tests were used for continuous variables to compare 2 groups with and without follow-up response.

^d^Not applicable.

^e^One responder with an outlier response (value of 1000) was excluded.

^f^PHQ-2: 2-item Patient Health Questionnaire.

^g^GAD-2: 2-item Generalized Anxiety Disorder scale.

^h^n=182.

^i^n=146.

^j^n=36.

^k^n=121.

^l^n=96.

^m^n=25.

^n^The inpatient or rehabilitation item in this set was assigned 0 if it was skipped but the item on other use was answered. The imputed version was for the sensitivity analysis.

^o^n=147.

^p^n=118.

^q^n=29.

Regarding the primary analyses, pretest-posttest reduction was significantly greater for LGBTQ+ participants than for non-LGBTQ+ participants in terms of both mean PHQ-2 score (−0.7, SD 1.7 vs −0.1, SD 1.4; t_180_=−2.16; *P*=.03) and mean GAD-2 score (−0.8, SD 2.0 vs 0.0, SD 1.2; t_177_=−3.08; *P*=.002). During the period before follow-up, LGBTQ+ participants reported greater use of behavioral health services than non-LGBTQ+ participants (16/26, 62% vs 37/96, 39%; χ^2^_1_=5.2; *P*=.02), including when imputing for missing data (16/29, 55% vs 37/118, 31.4%; χ^2^_1_=5.7; *P*=.02).

For the exploratory analyses, we examined which individual COVID-19 stressors differed significantly for LGBTQ+ and non-LGBTQ+ respondents at baseline and follow-up. At baseline, compared to non-LGBTQ+ participants, LGBTQ+ participants had (1) significantly greater depression during the COVID-19 pandemic (χ^2^_1_=4.0; *P*=.045); (2) increased alcohol or other substance use (χ^2^_1_=13.6; *P*<.001); (3) greater change in sexual activity (χ^2^_1_=13.6; *P*<.001); and (4) more financial support from family and friends (χ^2^_1_=9.2; *P*=.002), a potential indirect measure of financial difficulty or need. At follow-up, LGBTQ+ participants reported significantly greater (1) diagnoses of COVID-19 (χ^2^_1_=3.9; *P*=.05), (2) fear of giving COVID-19 to someone else (χ^2^_1_=10.0; *P*=.002), (3) frustration or boredom (χ^2^_1_=4.0; *P*=.047), (4) depression during the COVID-19 pandemic (χ^2^_1_=6.4; *P*=.01), (5) use of alcohol and other substances (χ^2^_10_=10.3; *P*=.001), (6) change in sexual activity (χ^2^_1_=4.6; *P*=.03), and (7) loneliness (χ^2^_1_=7.2; *P*=.007).

### Regression Analyses: Final Predictors of Site Use, Depression, and Anxiety at Follow-Up

Final significant predictors of follow-up measures, with adjustment for follow-up nonresponse (inverse probability weighting) for both LGBTQ+ and non-LGBTQ+ participants are provided in Table 3. For LGBTQ+ participants, final predictors of any visit to or use of the website or resources in the 4 to 6 weeks before follow-up included (1) baseline PHQ-2 or GAD-2 score of ≥3, which was significant when adjusting for nonresponse (odds ratio [OR] 5.75, 95% CI 1.13-29.43; *P*=.03); and (2) total COVID-19–related behavior changes, which was significant without and with adjusting for nonresponse (without adjusting: OR 0.59, 95% CI 0.36-0.98; *P*=.04; with adjusting: OR 0.62, 95% CI 0.40-0.98; *P*=.04). Predictors for moderate or greater depression (PHQ-2 score≥3) at follow-up were (1) baseline PHQ-2 score of ≥3 (which was significant when adjusting for nonresponse: OR 7.44, 95% CI 1.23-45.19; *P*=.03) and (2) having visited the T4W/Juntos website or used the resources before follow-up had a trend as a predictor of lower likelihood of depression, which was not significant without or with adjusting for nonresponse (without adjusting: OR 0.22, 95% CI 0.04-1.28; *P*=.09; with adjusting: OR 0.25, 95% CI 0.04-1.54; *P*=.13). Predictors of higher GAD-2 score at follow-up with and without adjusting for nonresponse were White ethnicity (with adjusting: OR 23.31, 95% CI 1.80-301.34; *P*=.02) and baseline GAD-2 score of ≥3 (with adjusting: OR 55.76, 95% CI 3.30-943.16; *P*=.005).

**Table 3:** Final models for predictors of visits to or use of the website and 2-item Patient Health Questionnaire (PHQ-2) score of ≥3 or 2-item Generalized Anxiety Disorder scale (GAD-2) score of ≥3 at follow-up (FU) and pretest-posttest mean score changes for the California Together for Wellness/Juntos por Nuestro Bienestar (T4W/Juntos) participants (April 2021-May 2022)—lesbian, gay, bisexual, transgender, queer, and questioning (LGBTQ+)^a^ and non-LGBTQ+^b^ baseline (BA) and FU predictors tested.

Variable				Main analysis (unweighted)		Sensitivity analysis (IPW^c^; nonresponse)	
				OR^d^ or β^e^ (95% CI)^e^	*P* value	OR or β (95% CI)	*P* value
**LGBTQ+ (not including the “don’t know” response option; n=37)**							
	**Predictors of any visit to or use of the website at follow-up^f^**						
		PHQ-2 or GAD-2 score of ≥3 (BA)		4.81 (0.92 to 25.27)^d^	.06	5.75 (1.13 to 29.43)^d^	.03
		Total number of COVID-19–related behavior changes (BA)		0.59 (0.36 to 0.98)^d^	.04	0.62 (0.40 to 0.98)^d^	.04
	**Secondary outcomes**						
		**Predictors of PHQ-2 score of ≥3 at follow-up^f^**					
			PHQ-2 score of ≥3 (BA)	6.62 (1.15 to 38.32)^d^	.04	7.44 (1.23 to 45.19)^d^	.03
			Any visit to T4W/Juntos website or use of resources (FU)	0.22 (0.04 to 1.28)^d^	.09	0.25 (0.04 to 1.54)^d^	.13
		**Predictors of GAD-2 score of ≥3 at follow-up^g^**					
			White ethnicity (BA)	29.67 (2.28 to 385.57)^d^	.01	23.31 (1.80 to 301.34)^d^	.02
			GAD-2 score of ≥3 (BA)	60.32 (3.35 to 1085.00)^d^	.005	55.76 (3.30 to 943.16)^d^	.005
	**Primary outcomes**						
		**Predictor of PHQ-2 pretest-posttest mean score change^f^**					
			Any visit to T4W/Juntos website or use of resources (FU)	−1.16 (−2.28 to 0.05)^e^	.04	−0.94 (−2.00 to 0.13)^e^	.09
		**Predictors of GAD-2 pretest-posttest mean score change^g^**					
			Hispanic or Latinx ethnicity (BA)	−1.68 (−3.22 to −0.14)^e^	.03	−1.95 (−3.20 to −0.70)^e^	.003
			Any visit to T4W/Juntos website or use of resources 4-6 wk before FU	−1.48 (−2.78 to −0.17)^e^	.03	−1.08 (−2.05 to −0.10)^e^	.03
**Female and male (binary) heterosexual participants (n=148)**							
	**Predictor of any visit to or use of the website at follow-up^h^**						
		Feeling comfortable using the website (BA)		0.43 (0.19 to 0.94)^d^	.03	0.44 (0.19 to 1.00)^d^	.05
	**Secondary outcomes**						
		**Predictors of PHQ-2 score of ≥3 at follow-up^h^**					
			PHQ-2 score of ≥3 (BA)	5.20 (1.83 to 14.72)^d^	.002	5.26 (1.72 to 16.10)^d^	.003
			Website engagement mean score (3 items^i^; BA)	0.55 (0.32 to 0.95)^d^	.03	0.55 (0.31 to 0.97)^d^	.04
		**Predictors of GAD-2 score of ≥3 at follow-up^j^**					
			GAD-2 score of ≥3 (BA)	21.21 (7.08 to 63.60)^d^	<.001	21.36 (6.34 to 71.94)^d^	<.001
			Total score of T4W/Juntos resource categories viewed or used (FU)	1.09 (1.01 to 1.18)^d^	.03	1.12 (1.12 to 1.23)^d^	.007
	**Primary outcomes**						
		**Predictors of PHQ-2 pretest-posttest mean score change^h^**					
			Any visit to T4W/Juntos website or use of resources (FU)	−0.46 (−0.93 to 0.00)^e^	.05	−0.37 (−0.84 to 0.10)^e^	.12
			Total number of COVID-19 stressors (BA)	−0.07 (−0.13 to −0.02)^e^	.009	−0.09 (−0.16 to −0.02)^e^	.02
		**Predictors of GAD-2 pretest-posttest mean score change^h^**					
			Age of 18-30 y (vs older; BA)	−0.58 (−0.97 to −0.19)^e^	.004	−0.55 (−0.95 to −0.16)^e^	.006
			High school educational level or lower (BA)	−0.80 (−1.28 to −0.39)^e^	<.001	−0.86 (−1.36 to −0.36)^e^	<.001
			Total number of COVID-19 stressors (BA)	−0.10 (−0.14 to −0.06)^e^	<.001	−0.10 (−0.16 to −0.05)^e^	<.001

^a^This category captures those with a gender identity other than male or female or a sexual orientation other than heterosexual (excluding missing values or “don’t know” or “prefer not to state” response options).

^b^The non-LGBTQ+ category includes participants who identified as heterosexual in their sexual orientation *and* with a binary gender identity (either male or female). None of these participants disclosed a transgender or gender-diverse identity.

^c^IPW: inverse probability weighting for nonresponse predictors at BA and FU.

^d^OR: odds ratio.

^e^β coeffient.

^f^Analytical sample: n=36.

^g^Analytical sample: n=33.

^h^Analytical sample: n=144.

^i^Items (ease of use, relevance of topics, and satisfaction) were averaged as mean engagement based on 5-point Likert scales.

^j^Analytical sample: n=139.

For the non-LGBTQ+ sample (Table 3), the significant predictor of use of the website or resources before follow-up with and without adjusting for nonresponse was baseline comfort with using the website (without adjusting: OR 0.43, 95% CI 0.19-0.94; *P*=.03; with adjusting: OR 0.44, 95% CI 0.19-1.00; *P*=.05). The total score of website use before follow-up was a significant predictor of a GAD-2 score of ≥3 before follow-up with and without adjusting for nonresponse (without adjusting: OR 1.09, 95% CI 1.01-1.1.8; *P*=.03; with adjusting: OR 1.12, 95% CI 1.12-1.23; *P*=.007). Mean website engagement was a significant predictor of a PHQ-2 score of ≥3 before follow-up with and without adjusting for nonresponse (without adjusting: OR 0.55, 95% CI 0.32-0.95; *P*=.03; with adjusting: OR 0.55, 95% CI 0.31-0.97; *P*=.04).

Regarding the primary outcomes of pretest-posttest reduction (Table 3) for LGBTQ+, for mean PHQ-2 score, the significant predictor was having visited the T4W/Juntos website or used the resources 4 to 6 weeks before follow-up (β=−1.16, 95% CI −2.28 to 0.05; *P*=.04), but it was not significant at *P*<.05 when adjusting for nonresponse (β=−0.92, 95% CI −2.00 to 0.13; *P*=.09). Regarding pretest-posttest reduction in GAD-2 scores, significant predictors without and with adjusting for nonresponse included Hispanic or Latinx ethnicity at baseline (without adjusting: β=−1.68, 95% CI −3.22 to −0.14; *P*=.03; with adjusting: β=−1.95, 95% CI −3.20 to −0.70; *P*=.003) and having visited the website or used the resources before follow-up (without adjusting: β=−1.48, 95% CI −2.78 to −0.17; *P*=.03; with adjusting: β=−1.08, 95% CI −2.05 to –0.10; *P*=.03).

Regarding the main exploratory outcomes (Table 3) for non-LGBTQ+, for pretest-posttest change in PHQ-2 score, having visited the website or used the resources before follow-up had a trend as predictor of reduction in depression (β=−0.046, 95% CI −0.93 to 0.00; P=.05), which was not significant when adjusting for nonresponse (β=−0.37, 95% CI −0.84 to 0.10; P=.12). For pretest-posttest change in GAD-2 score, this website use variable was not in the final models given that it was not significant in the initial models (t_142_=−0.56; *P*=.58), but rather younger age, lower education, and COVID-19 stressors were included as significant predictors (Table 3).

## Discussion

### Principal Findings and Comparison With Prior Work

This exploratory subanalysis focused on the experiences of LGBTQ+ adults (aged ≥18 years) in California and in comparison to non-LGBTQ+ adults who completed baseline and 4- to 6-week follow-up surveys after visiting a free mental well-being website during the COVID-19 pandemic.

LGBTQ+ participants exhibited greater baseline depression and anxiety than non-LGBTQ+ participants, consistent with previous literature on long-term LGBTQ+ mental health inequities [1-11] and COVID-19–associated mental health inequities [12-18,65]. In 3-way comparisons, LGBTQ+ participants had greater baseline depression than female heterosexual participants and greater anxiety than both female and male heterosexual participants. LGBTQ+ participants reported more COVID-19 stressors and higher use of behavioral health services than non-LGBTQ+ participants at baseline and follow-up. Collectively, these findings support those of previous studies on minority stress among LGBTQ+ populations, suggesting that mental health inequities stem from social stressors such as discrimination and their impacts on internalized expectations, self-efficacy, and self-image [6,7]. To more fully understand this, we explored differences in individual COVID-19 stressors, finding that LGBTQ+ participants exhibited a higher number of stressors than non-LGBTQ+ participants in 4 areas at baseline (depression, increased alcohol use, change in sexual behavior, and increased financial support from others) and in 7 areas at follow-up (the first 3 mentioned previously plus being diagnosed with COVID-19, fear of giving it to someone, frustration or boredom, and loneliness). Such findings mirror those of studies illustrating disproportionate burden and may suggest specific areas of support needed for LGBTQ+ communities [20-22].

Regarding the main analyses, we found that LGBTQ+ participants had a greater pretest-posttest decline in depression and anxiety than non-LGBTQ+ participants, and in 3-way comparisons for the sensitivity analysis (Multimedia Appendix 1), LGBTQ+ participants had a greater reduction in anxiety than female heterosexual participants. Before follow-up, LGBTQ+ participants had a higher behavioral health service use than non-LGBTQ+ participants, which was significant when imputing for missing data and for LGBTQ+ participants compared to female heterosexual participants in 3-way comparisons. This suggests a greater reduction in anxiety over time for LGBTQ+ participants after initially presenting with higher anxiety levels while having a higher use of behavioral health services between baseline and follow-up.

To understand potential explanatory mechanisms, we used sequential regression analyses to find the best predictors of follow-up website use, depression and anxiety at follow-up, and changes over time in depression and anxiety. For LGBTQ+ participants, predictors of visiting the website or using website resources were depression or anxiety before baseline (which was significant when imputing for missing data) and total COVID-19–related behavior changes, suggesting that persons with higher need were more likely to visit the website or use the resources. Regarding primary analyses of predictors of pretest-posttest reduction in depression (PHQ-2), the main predictor was visiting the website before follow-up, which was significant in the main analysis but borderline significant when imputing for missing data. For reduction in anxiety (GAD-2), website use and Hispanic or Latinx ethnicity were significant predictors.

For non-LGBTQ+ participants, the main predictor of website use was comfort using the website, and total website use and engagement was associated with follow-up depression and anxiety. For pretest-posttest change in need, having visited the website was not significant at *P*<.05 for reduction in PHQ-2 scores, including when adjusting for missing data, and website use did not predict reduction in GAD-2 scores. Similarly, in the sensitivity analyses for female and male heterosexual participants separately (Multimedia Appendix 1), website use did not predict reduction in depression or anxiety. Thus, the association between reduction in anxiety and website use was primarily for LGBTQ+ adults.

### Limitations

This evaluation focused on participants invited via emails from partnering agencies that participated in developing the website, which could mean that participants might feel more positive about the website. It is possible that those who agreed to participate may also have had higher digital (computer or internet) access or familiarity than others.

LGBTQ+ participants represented approximately 20% of our sample (64/315, 20.3%), which fits with data on US younger adult populations (millennials, born between 1981 and 1996 and younger) [66]. While consistent with general populations, the overall sample size of LGBTQ+ participants (64/315, 20.3%; 37/64, 58% with follow-up survey data) was modest, and most participants identified as female and heterosexual (225/315, 71.4% and 236/315, 74.9%, respectively). Three-way comparisons among LGBTQ+, male heterosexual, and female heterosexual participants were included for sensitivity analyses in Multimedia Appendix 1. The sample in this study was mainly binary in its distribution of gender identities, with only 4.8% (15/315) of the respondents identifying as gender diverse (including genderqueer, questioning, transgender men, transgender women, or other gender). Among millennials, approximately 12% identify as gender diverse (including transgender) [66]. Our gender identity data may have limitations, consistent with studies showing diversity in how transgender and nonbinary adults describe their gender identities [67] as some may not disclose due to concerns about privacy, discrimination, or other factors [48,50].

Furthermore, to capture LGBTQ+ identities, we used 2 measures: sexual orientation and gender identity, consistent with the NIH 2022 recommendations [48]. With partner input, we used one measure of gender identity, not including sex assigned at birth, and did not capture multiple dimensions of sexual orientation recommended by others (eg, romantic attraction, sexual attraction, and sexual behavior) [47]. For the analyses, we excluded participants who did not provide information on gender identity and sexual orientation—some of whom may have been LGBTQ+. However, all gender identity and sexual orientation survey categories assessed had at least one representative in the follow-up sample. For the main regression analyses exploring predictors of outcomes, the subsample size of LGBTQ+ individuals who had pre- and posttest data was modest (n=30-34), and while key findings about the effects of website use were statistically significant, it will be important to conduct larger studies in the future to confirm such effects in this context.

Other limitations include that the study used baseline and follow-up surveys without intervention comparison groups or randomization; thus, predictors of outcomes may not clearly represent causation. For example, the association between greater website use and reduced anxiety before follow-up could reflect the effects of website use or suggest that those with declining anxiety were more likely to use the website. As a formative and exploratory study, we did not formally adjust for multiple comparisons, but for the hypothesized greater depression and anxiety and higher number of COVID-19 stressors for LGBTQ+ participants than for non-LGBTQ+ participants at baseline, significance ranged from *P*=.005 to *P*<.001, so it was significant with multiple comparisons. Regarding the association between website use and decline over time in mean depression and anxiety for LGBTQ+ participants, significance ranged from *P*=.04 to *P*=.09 for depression and *P*=.03 for anxiety; thus, adjusting for 2 comparisons, the results are considered exploratory to inform future research.

### Public Health Implications

Overall, our findings showed that LGBTQ+ participants in our sample of community-recruited adults who visited a public well-being website and provided survey data had greater depression and anxiety and more COVID-19 stressors than non-LGBTQ+ participants. Over time, LGBTQ+ participants exhibited a greater reduction in anxiety than non-LGBTQ+ participants and, separately, than female heterosexual and male heterosexual participants. Specific factors such as COVID-19 stressors (eg, loss and financial strain) may be associated with increased baseline depression and anxiety in this population. Knowing specific risk factors and stressors may inform efforts to mobilize relevant resources to LGBTQ+ populations during pandemics. Consistent with this hypothesis, LGBTQ+ participants had a more consistent prediction of reduced anxiety over time associated with website use, which was not a predictor for non-LGBTQ+ participants. These findings, though exploratory, suggest that digital resources created with community partner input [38] and consistent with community-partnered participatory research, the technology acceptance model, and the vulnerable populations framework [38,41,43] may be helpful when tailored to LGBTQ+ populations—or potentially other communities facing great stressors or mental health symptoms. These are important areas for further research that may inform practice and policy strategies on digital interventions to support well-being in LGBTQ+ during crises such as COVID-19.

## Appendix

Multimedia Appendix 1 Sensitivity analyses (3-category comparisons for LGTBQ+, female heterosexual and male heterosexual).

## Abbreviations

GAD-2: 2-item Generalized Anxiety Disorder scale
LGBTQ+: lesbian, gay, bisexual, transgender, queer, and questioning
NIH: National Institutes of Health
OR: odds ratio
PHQ-2: 2-item Patient Health Questionnaire
T4W/Juntos: Together for Wellness/Juntos por Nuestro Bienestar

## Data Availability

The datasets generated or analyzed during this study are available from the corresponding or statistician authors on reasonable request.
