# Supplementary material for: Exploring the Impact of Online Mental Health Resources During the COVID-19 Pandemic on Lesbian, Gay, Bisexual, Transgender, Queer, and Questioning Adults Compared to Heterosexual Adults: Pretest-Posttest Survey Analyses
Source: JMIR Form Res. 2025 Jul 11;9:e67082. doi: 10.2196/67082 (PMC12299941; doi:10.2196/67082)
Supplement: Multimedia Appendix 1 [file formative_v9i1e67082_app1.doc]

*Appendix: Sensitivity Analyses (3-Category Comparisons for LGTBQ+, Female Heterosexual and Male Heterosexual)*

*This Appendix reviews the findings for analyses when female and male heterosexual are separate categories and in comparison to LGTBQ+, with Appendix Tables 1-4.*

*Baseline:* Of the total analysis sample (N=300), 183 identified as female heterosexual and 53 as male heterosexual (Appendix Table 1). For three-category sensitivity analysis (LGBTQ+, female heterosexual and male heterosexual), overall comparison of 3 groups was significant at baseline for: (1) mean age, (*F_2,299_*=9.71, *P*<.001), with 2-way comparison significant for LGBTQ+ younger than female heterosexual (mean 33.1 (SD 12.4) versus mean 41.4 (SD 13.5), *t_245_*=4.3, *P*<.001); 2) race/ethnicity (*χ*^2^*_6_*=13.41, *P*=.04); with 2-way comparisons significant for female versus male heterosexual (*χ*^2^*_3_*=10.44, *P*=.045) and female lower percent white/Caucasian, 20.9% (37/177) versus male 42.0% (21/50) and higher percent Hispanic, 50.3% (98/177) versus 30.0% (15/50), respectively; (3) language preference, (*χ*^2^*_4_*=10.38, *P*=.03), with no significant 2-way comparisons (each *P*>.05); 4) mean PHQ-2 (*F_2,296_*=14.39, *P*<.001), with LGBTQ+ higher (2.4, SD 1.7), than female heterosexual (1.3, SD 1.3) *t_243_*=5.24, *P*<.001, and than male heterosexual (1.5, SD 1.2) (*t_111_*=3.21, *P*=.005); (4) mean GAD-2 (*F_2,296_*=12.31, *P*<.001), with LGBTQ+ higher anxiety (2.7 (SD 1.7) than female heterosexual (1.5, SD 1.5), *t_244_*=4.87, *P*<.001 and than male heterosexual (1.6, SD 1.6) *t_112_*= -3.39, *P*=.003; (5) use of behavioral health services (main, *χ*^2^*_2_*=11.3, *P*=.004; with imputation for nonresponse *χ*^2^*_2_*=12.8, *P*=.002); with greater use for LGBTQ+, without imputation 33/41 (80.5%); with imputation 33/49 (68.8%) compared to female heterosexual, without imputation 59/113 (52.2%); with imputation 50/143 (41.3%), each significant (without adjustment, *χ*^2^_1_=10.0, *P*=.005; with imputation, *χ*^2^*_1_*=10.88, *P*=.003); (6) COVID-19 stressors (*F_2,299_*=4.21, *P*=.02), with more stressors for LGBTQ+, mean 8.2 (SD 4.4) than for female heterosexual, mean 6.6 (SD 4.0), *t_245_*=2.51, *P*=.04, and for male heterosexual, mean 6.1 (SD 3.9), *t_115_* =-2.56, *P*=.04.

*Follow-up:* For follow-up nonresponse (Appendix Table 2), for three-group comparison, overall difference was significant (*χ*^2^*_2_*=10.32, *P*=.006), and for two-way comparisons, nonresponse was significantly lower for male heterosexual (10/53, 18.9%) versus female heterosexual (78/183, 42.6%) *χ*^2^*_1_* =9.92, *P*=.002); and versus LGBTQ+ (27/64, 42.2%) *χ*^2^*_1_* =6.97, *P*=.008).

For three-category at follow-up (Appendix Table 3), overall comparison was significant for: (1) visited website or used resources 4-6 weeks prior (*χ*^2^*_2_*=8.75, *P*=.01), with higher percentage use for heterosexual male (79.1%, 34/43) than heterosexual female (53.3%, 56/105), *χ*^2^*_2_*=8.48, *P*=.01. The overall 3-way comparison was not significant for total score of T4W/Juntos resources used (*F_2,177_*=1.28, *P*=.28); for recommending the website to others (*F_2_*,*_92_*=2.07, *P*=.36), or for those recommending, mean number of times (*F_2,176_*=1.99, *P*=.14). The 3-way comparison was significant for mean PHQ-2 (F_[2,184]_=3.33, p=.04), but without significant two-way comparisons (each *P*>.05); and 3-way was significant for PHQ-2>=3 (*χ*^2^*_2_*=7.54, *P*=.02), with greater probability of depression for LGBTQ+ (13/37, 35.1%) than female heterosexual (15/105, 14.3%),

*χ*^2^*_1_*=8.78, *P*=.009. The 3-way comparison was significant for post-pre reduction in mean GAD-2 (*F*_2,178_ =4.76, *P*=.01), with greater reduction in anxiety for LGBTQ+, -0.8 (SD 2.0) than female heterosexual, 0.0 (SD 1.2), t*_138_* =2.9, *P*=.01; and 3-way comparison for reduction in post-pre mean PHQ-2 was not significant (F_2,181_=2.86, *P*=0.06) with no significant two-way comparisons. The 3-category comparison of follow-up mean mental health stigma score was significant (F_2,178_ =4.08, *P*=.02) with higher stigma for male, 3.3 (SD 1.0) versus female, 2.8 (SD 0.9) heterosexual (*t_143_* =2.81, *P*=.02). For services use for behavioral health, overall 3-way comparison was not significant (*χ*^2^*_2_*=5.63, *P*=.06), but significant imputing for missing data (*χ*^2^*_2_*=7.01, *P*=.03), with higher use by LGBTQ+ (16/29, 55.2%) than female heterosexual (24/28.2, 28.2%), *χ*^2^*_1_*=7.81, *P*=.02. For total COVID-stressors, 3-way comparison was significant (*F_2,184_*=3.63, *P*=.03) with LGBTQ+ higher stressors, 8.4 (SD 4.4) than male heterosexual, 6.0 (SD 4.1) t*_79_*= -2.77, *P*=.02.

*Regression Models for post-pre reduction in depression and anxiety*: For male and female heterosexual groups separately, no final models had website use as a significant predictor of post-pre change in mean PHQ2 or GAD2 (each p>.05) (Appendix Table 4).

*Appendix Tables 1-4*

|  | *Appendix Table 1:* Characteristics of study participants, LGBTQ+^a^ versus female or male heterosexual, baseline (9/21-4/22) includes two-way comparisons of three groups) for California T4W/Juntos website study | | | | | | | |
| --- | --- | --- | --- | --- | --- | --- | --- | --- |
| Characteristics (Baseline) | | | Overall  (N=300) | LGBTQ+  (N=64, 21.3%) | Female  Heterosexual  (N=183, 61.0%) | Male  Heterosexual  (N=53, 17.7%) | Overall Statistic  *F* (df) or *Chi-square* (Chis) (*df*)  (p value)^b^ | 2-way:^c^ t-test (df) or Chis (df)  A:LGBTQ+ vs. female heterosexual  B:LGBTQ+ vs. male heterosexual  C: female vs. male heterosexual |
| Age (years), N, mean (SD) | | | 300, 38.9 (13.7) | 64, 33.1 (12.4) | 183, 41.4 (13.5) | 53, 37.1 (13.6) | 9.71 (2, 299) *P*<.001 | A: t (245) =4.3, *P*<.001  B: t (115) = 1.68, *P*=.29  C: t (234) = -2.01,*P*=.14 |
|  | Race, n (%) | | | | | |  |  |
|  | | Responses, N | 288 | 61 | 177 | 50 | 13.41 (6)  *P* =.04 | A:Chis(3)=6.16,*P*=.31  B:Chis(3) =1.11, *P*>.99  C:*Chis(3)* =10.44, *P*=.045 |
|  | | Hispanic | 125 (43.4) | 21 (34.4) | 89 (50.3) | 15 (30.0) |  |  |
|  | | Black/African American | 53 (18.4) | 13 (21.3) | 32 (18.1) | 8 (16.0) |  |  |
|  | | White/Caucasian | 79 (27.4) | 21 (34.4) | 37 (20.9) | 21 (42.0) |  |  |
|  | | Other | 31 (10.8) | 6 (9.8) | 19 (10.7) | 6 (12.0) |  |  |
|  | Education, n (%) | | | | | |  |  |
|  | | Responses, n | 298 | 63 | 182 | 34 | 8.78 (8)  *P* =.36 | A:Chis(4)=.26, *P*>.99  B:Chis(4)=5.83, *P*=.64  C:Chis(4)=7.87, *P*=.29 |
|  | | Less than high school | 21 (7.0) | 5 (7.9) | 12 (6.6) | 4 (7.5) |  |  |
|  | | High school graduate | 34 (11.4) | 6 (9.5) | 19 (10.4) | 9 (17.0) |  |  |
|  | | Some college | 84 (28.2) | 16 (25.4) | 47 (25.8) | 21 (39.6) |  |  |
|  | | College | 113 (37.9) | 26 (39.7) | 75 (41.2) | 13 (24.5) |  |  |
|  | | Graduate school | 46 (15.4) | 11 (17.5) | 29 (15.9) | 6 (11.3) |  |  |
|  | Language prefer to use on the website, n (%) | | | | | |  |  |
|  | | Responses, n | 298 | 63 | 182 | 53 | 10.38 (4)  *P* =.03 | A:Chis(2)=7.4, *P*=.07  B:Chis(2)=3.67, *P*=.48  C:Chis(2)=3.68, *P*=.48 |
|  | | English | 250 (83.9) | 59 (93.7) | 144 (79.1) | 47 (88.7) |  |  |
|  | | Spanish | 36 (12.1) | 4 (6.3) | 29 (15.9) | 3 (5.7) |  |  |
|  | | Other | 12 (4.0) | 0 (0.0) | 9 (4.9) | 3 (5.7) |  |  |
| PHQ-2 score, N, mean (SD) | | | 296, 1.6 (1.5) | 62, 2.4 (1.7) | 183, 1.3 (1.3) | 51, 1.5 (1.2) | 14.39 (2, 296) *P*<.001 | A: t (243)=-5.24, *P*<.001  B: t(111)= -3.21, *P*=.005  C: t(232)= .81, *P*>.99 |
|  | PHQ-2≥3, n (%) | | | | | |  |  |
|  | | Responses, n=297 | 296 | 63 | 183 | 51 | 23.86 (2)  *P* <.001 | A: Chis (1) =23.92, *P*<.001  B: Chis (1)=5.62, *P*=.053  C: Chis (1)=1.9, *P*=.49 |
|  | | Yes | 58 (19.5) | 26 (41.3) | 22 (12.0) | 10 (19.6) |  |  |
|  | | No | 239 (80.5) | 37 (58.7) | 161 (88.0) | 41 (80.4) |  |  |
| GAD-2 score, N, mean (SD)   | t_[232]_=0.3 | 1 | | --- | --- | | | | 297, 1.8 (1.7) | 63, 2.7 (1.7) | 183, 1.5 (1.5) | 51, 1.6 (1.6) | 12.31 (2, 296) *P*<.001 | A: t (244) =-4.87, *P*<.001  B: t (112)= -3.39, *P*=.003  C: t (232)= .3, *P*>.99 |
|  | GAD-2≥3, n (%) | | | | | |  |  |
|  | | Responses, n | 297 | 63 | 183 | 51 | 23.51 (2) *P*<.001 | A: *Chis* (1*)*=20.63, *P*<.001  B: *Chis*(1*)* =12.32, *P*=.001  C: Chis (1)=.11, *P*>.99 |
|  | | Yes | 76 (25.6) | 31 (49.2) | 36 (19.7) | 9 (17.6) |  |  |
|  | | No | 221 (74.4) | 32 (50.8) | 147 (80.3) | 42 (82.4) |  |  |
| Stigma score, N, mean (SD) | | | 277, 2.8 (1.0) | 61, 3.0 (0.9) | 169, 2.7 (1.0) | 47, 3.1 (0.9) | 3.75 (2, 276) *P*=.03 | A:t(228)= -1.99, *P*=.145  B:t(106)=.44, *P*>.99  C:t(214)=2.27, *P*=.07 |
| Engagement score (3 items)^f^, N, mean (SD) | | | 290, 4.0 (0.7) | 60, 4.0 (0.7) | 178, 4.1 (0.7) | 52, 3.9 (0.7) | 2.02 (2, 289) *P*=.14 | A:t(236)=1.141, *P*=.466  B;t(110)= -.33, *P*>.99  C:t(228)= -1.71, *P*=.14 |
|  | Do not feel comfortable using this website, n (%) | | | | | |  |  |
|  | | Responses, n | 291 | 62 | 178 | 51 | 10.17 (8) *P*=.26 | A:Chis (4)=2.72, *P*>.99  B:Chis (4)=5.39, *P*=.75  C:Chis (4)=6.54, *P*=.49 |
|  | | Strongly disagree | 99 (34.0) | 25 (40.3) | 60 (33.7) | 14 (27.5) |  |  |
|  | | Disagree | 105 (36.1) | 24 (38.7) | 64 (36.0) | 17 (33.3) |  |  |
|  | | Neither agree nor disagree | 22 (7.6) | 3 (4.8) | 16 (9.0) | 3 (5.9) |  |  |
|  | | Agree | 36 (12.4) | 6 (9.7) | 18 (10.1) | 12 (23.5) |  |  |
|  | | Strongly agree | 29 (10.0) | 4 (6.5) | 20 (11.2) | 5 (9.8) |  |  |
|  | Any service use for emotional, mental health, alcohol, or drug problems, n (%) | | | | | |  |  |
|  | | Responses, n | 187 | 41 | 113 | 33 | 11.3 (2) *P*=.004 | A: Chis (1)=10.0, *P* =.005  B:Chis (1)=1.16, *P*=.85  C: Chis (1)=3.17, *P*=.23 |
|  | | Yes | 115 (61.5) | 33 (80.5) | 59 (52.2) | 23 (69.7) |  |  |
|  | | No | 72 (38.5) | 8 (19.5) | 54 (47.8) | 10 (30.3) |  |  |
|  | Any service use for emotional, mental health, alcohol, or drug problems, imputed^g^, n (%) | | | | | |  |  |
|  | | Responses, n | 229 | 49 | 143 | 38 | 12.8 (2)  *P* =.002 | A:Chis(1)=10.88, *P* =.003  B: Chis (1)=.63, *P*>.99  C:Chis (1) =4.5, *P*=.10 |
|  | | Yes | 115 (50.2) | 33 (68.8) | 59 (41.3) | 23 (60.5) |  |  |
|  | | No | 114 (49.8) | 15 (31.3) | 84 (58.7) | 15 (39.5) |  |  |
| Total number of COVID-19–related behavior changes, N, mean (SD) | | | 300, 3.9 (2.0) | 64, 4.0 (2.2) | 183, 3.9 (1.9) | 53, 3.5 (1.9) | 1.33 (2, 299) *P* =.27 | A: t(245)= -.52, *P*>.99  B: t(115)= -1.51, *P*=.40  C: t(234)= -1.41, *P*=.48 |
| Total number of COVID-19 stressors experienced, N, mean (SD) | | | 300, 6.9 (4.1) | 64, 8.1 (4.4) | 183, 6.6 (4.0) | 53, 6.1 (3.9) | 4.21 (2, 299) *P* = .02 | A: t(245)=-2.51, *P* =.04  B: t(115)=-2.56, *P* =.04  C: t(234)= -.79, *P*>.99 |
|  | ^a^LGBTQ+ stands for lesbian, gay, bisexual, transgender, and queer/questioning; category reflects those with a gender identity other than male or female and/or a sexual orientation other than heterosexual (excluding missing/don’t know/prefer not to state)  female, male heterosexual;  ^b^Chi-square tests (Chis) (df) were used for categorical variables and F-tests (df) were for continuous variables to compare 3 groups.  ^c^Two-way comparison (A=female heterosexual versus LGBTQ+; B=male heterosexual versus LGBTQ+; C=female heterosexual versus male heterosexual)  ^d^PHQ-2: Patient Health Questionnaire-2 item.  ^e^GAD-2: Generalized Anxiety Disorder-2 item scale.  ^f^Items (ease of use, relevance of topics, satisfaction) were averaged as mean engagement based on 5-point Likert scales.  ^g^Inpatient/rehabilitation item in this set was assigned 0 if the item was skipped but other use item answered. Imputed version is for sensitivity analysis. | | | | | | | |

| **Appendix Table 2:** Follow-up survey completion (10/21-5/22) by participant status at baseline (9/21-4/22) of LGBTQ^a^ versus female and male heterosexual, for California T4W/Juntos website study | | | | | | |
| --- | --- | --- | --- | --- | --- | --- |
| Sexual orientation and/or gender identity Category (Baseline) | | Overall  (n=315) | No follow-up  (n=122, 23.5%) | Follow-up  (n=193,61.3%) | Statistic  *Chi-square* (*df*)^b^ | *P* value |
| Sexual orientation and gender identity not including don’t know (3 categories) Analytic N=300^c^ | |  |  |  | 10.32 (2) | .006 |
|  | LGBTQ+ | 64 (21.3) | 27 (42.2) | 37 (57.8) |  |  |
|  | Female heterosexual | 183 (61.0) | 78 (42.6) | 105 (57.4) |  |  |
|  | Male heterosexual | 53 (17.7) | 10 (18.9) | 43 (81.1) |  |  |
| ^a^LGBTQ+ stands for lesbian, gay, bisexual, transgender, and queer/questioning; category reflects those with a gender identity other than male or female and/or a sexual orientation other than heterosexual (excluding missing/don’t know/prefer not to state)  female, male heterosexual;  ^b^Chi-square tests (Chis) (df) used for categorical variables to compare two or three groups with and without follow up response. | | | | | | |
| ^c^2-way comparisons: LGBTQ+ vs. Male heterosexual, Chis (2) =6.97, *P*=.0083; Female heterosexual vs Male heterosexual Chis (2) =9.92, *P*=.0016); LGBTQ+ vs. Female heterosexual not significant (p>.05) | | | | | | |

| **c** | *Appendix Table 3:* Characteristics of participants, LGBTQ^+a^ versus female and male heterosexual, follow-up (10/21-5/22) (3-way comparison, and significant 2-way comparisons) for CA T4W/Juntos website study | | | | | | | |
| --- | --- | --- | --- | --- | --- | --- | --- | --- |
| Characteristics (Follow-up) | | | Overall  (N=185) | LGBTQ+  (n=37,20.0%) | Female Straight (n=105, 56.8%) | Male Straight  (n=43, 23.2%) | Overall Statistic  *F* or Chi-Square (Chis) (*df*)^b^ *P* value | 2-way:  A:LGBTQ+ vs. female heterosexual  B:LGBTQ+ vs. male heterosexual  C: female vs. male heterosexual |
| Visited T4W/Juntos site or used resources, 4-6 prior | | | | | | | | |
|  | | Responses, n | 185 | 37 | 105 | 43 | 8.75 (2) *P*=.013 | A: Chis(1)=1.76,*P*=.55  B: Chis(1)=1.8,*P*=.54  C:Chis(1)=8.48,*P*=.01 |
|  | | Yes | 114 (61.6) | 24 (64.9) | 56 (53.3) | 34 (79.1) |  |  |
|  | | No | 71 (38.4) | 13 (35.1) | 49 (46.7) | 9 (20.9) |  |  |
| Total score of T4W/Juntos resource categories viewed/used 4-6 weeks prior, N, mean (SD) | | | 178, 11.9 (6.5) | 36, 12.9 (7.7) | 102, 11.2 (6.5) | 40, 12.6 (5.2) | 1.28 (2, 177) *P*=.279 | A: t(137)= -1.51, *P*=.40  B: t(75)= -.38, *P*>.99  C: t(140)=1.23, *P*=.663 |
| Recommend the website to others (4-6 weeks prior) | | | | | | | | |
|  | | Responses, n | 185 | 37 | 105 | 43 | 2.07 (92) *P*=.355 | A:Chis(1)=1.46, *P*=.679  B:Chis(1)=.08, *P*>.99  C:Chis(1)=.82, *P*>.99 |
|  | | Yes | 110 (59.5) | 19 (51.4) | 67 (63.8) | 24 (55.8) |  |  |
|  | | No | 75 (40.5) | 18 (48.6) | 38 (36.2) | 19 (44.2) |  |  |
| Number of times recommended^c^, N, mean (SD) | | | 176, 3.0 (4.6) | 35, 3.1 (5.3) | 99, 3.3 (4.7) | 42, 2.2 (3.4) | 0.79 (2, 175)  *P*=.45 | A:t(132)= 0.25,*P*>.99  B:t(75)= -0.82, *P*>.99  C:t(139)= -1.32, *P*=.57 |
| Follow-up Mental Health | | | | | | | | |
| PHQ-2 score, N, mean (SD) | | | 185, 1.5 (1.4) | 37, 1.8 (1.6) | 105, 1.3 (1.3) | 43, 1.7 (1.2) | 3.33 (2, 184) *P*=.02 | A:t(141)=-2.52,*P*=.04  B:t(79)= -.69, *P*>.99  C:t(146)=1.99, *P*=.15 |
| PHQ-2≥3 (High), n (%) | | | | | | | | |
|  | | Responses, n | 185 | 37 | 105 | 43 | 7.54 (2) *P* =.02 | A:Chis(1) =8.78, *P*=.009  B:Chis(1)=1.79, *P*=.54  C:Chis(1)=1.75, *P*=.56 |
|  | | High | 38 (20.5) | 13 (35.1) | 15 (14.3) | 10 (23.3) |  |  |
|  | | Low | 147 (79.5) | 24 (64.9) | 90 (85.7) | 33 (76.7) |  |  |
| PHQ2 post-pre change score, N, mean (SD) | | | 182, -0.2 (1.5) | 36,-0.7 (1.7) | 105, -0.2 (1.4) | 41, 0.1 (1.5) | 2.86 (2, 181) *P*=.06 | A:t(140)=1.77, *P*=.24  B:t(76)=2.16, *P*=.10  C:t(144)=1.05, *P*=.89 |
| GAD-2 score, N, mean (SD) | | | 182, 1.7 (1.6) | 36, 2.1 (2.0) | 104, 1.7 (1.6) | 42, 1.5 (1.4) | 1.17 (2,182) *P*=.31 | A:t(139)= -1.47, *P*=.43  B:t(77)= -1.67, *P*=0.30  C:t(144)= -.66, *P*>.99 |
| GAD-2≥3 (High), n (%) | | | | | | | | |
|  | | Responses, n | 182 | 36 | 104 | 42 | 2.44 (2) *P*=.30 | A:Chis(1)=2.28, *P*=.39  B:Chis(1)=2.68, *P*=.31  C:Chis(1)=.24, *P*>.99 |
|  | | Yes | 39 (21.4) | 11 (30.6) | 21 (20.2) | 7 (16.7) |  |  |
|  | | No | 143 (78.6) | 25 (69.4) | 83 (79.8) | 35 (83.3) |  |  |
| GAD-2 post-pre change score, N, mean (SD) | | | 179, -0.2 (1.4) | 35, -0.8 (2.0) | 104,0.0 (1.2) | 40, -0.1 (1.2) | 4.76 (2,178) *P*=.01 | A: t(138)=2.9, *P*=.01  B:t(74)=1.99, *P*=.15  C:t(142)= -.35, *P*>.99 |
| Stigma score, N, mean (SD) | | | 179, 2.9 (1.0) | 34, 2.8 (1.1) | 103,2.8 (0.9) | 42, 3.3 (1.0) | 4.08 (2,178) *P*=.02 | A:t(136)=.03, *P*>.99  B:t(75)=2.05, *P*=.13  C: t(143)=2.81, *P*=02 |
| Any service use for emotional, mental health, alcohol, or drug problems, n (%) | | | | | | | | |
|  | | Responses, n | 121 | 25 | 66 | 30 | 5.63 (2) *P*=.06 | A:Chis(1)=6.36, *P*=.04  B:Chis(1)=2.72, *P*=.30  C:Chis(1)=.42, *P*>.99 |
|  | | Yes | 53 (43.8) | 16 (64.0) | 24 (36.4) | 13 (43.3) |  |  |
|  | | No | 68 (56.2) | 9 (36.0) | 42 (63.6) | 17 (56.7) |  |  |
| Any service use for emotional, mental health, alcohol, or drug problems, imputed^f^, n (%) | | | | | | | | |
|  | | Responses, n | 147 | 29 | 85 | 33 | 7.01 (2) *P*=.03 | A: Chis(1)=7.81, *P*=.02  B: Chis(1)=1.88, *P*=.51  C: Chis(1)=1.38, *P*=.72 |
|  | | Yes | 53 (36.1) | 16 (55.2) | 24 (28.2) | 13 (39.4) |  |  |
|  | | No | 94 (63.9) | 13 (44.8) | 61 (71.8) | 20 (60.6) |  |  |
| Total number of COVID-19 stressors experienced, N, mean (SD) | | | 185,7.0 (4.3) | 37, 8.5 (4.4) | 105, 6.9 (4.2) | 43, 6.0 (4.1) | 3.63 (2, 184) *P*=.03 | A: t(141)= -2.12, *P*=.11  B: t(79)=-2.77, *P*=.02  C: t(146)= -1.2, *P*=.70 |
|  | ^a^LGBTQ+ sexual preference other than straight and/or gender identity other than male or female (not including missing/don’t know/prefer not to state); straight/heterosexual female, male (selected gender identity and sexual identity).  ^b^Chi-square (Chis) (df) tests were used for categorical variables and t-tests (df) were used for continuous variables to compare two groups with and without follow up response.  ^c^one responder with outlier response (=1000) was excluded  ^d^PHQ-2: Patient Health Questionnaire-2 item.  ^e^GAD-2: Generalized Anxiety Disorder-2 item scale.  ^f^Inpatient/rehabilitation item was assigned 0 if the item was skipped but other use item answered. Imputed version is for sensitivity analysis. | | | | | | | |

| *Appendix Table 4.* Final models, CA T4W/Juntos website study, for Follow-up Visited/Used Website PHQ2>=3 or GAD2>=3 and post-pre Mean scores for Female and Male Heterosexual Samples with Baseline (BA, 9/21-4/22) and Follow-up (FU, 10/21-5/33) predictors tested | | | | | | | |
| --- | --- | --- | --- | --- | --- | --- | --- |
| Variables | | Main analysis (unweighted) | | | Sensitivity analysis (IPW^a^, nonresponse) | | |
|  | | Statistic^b^ | 95% CI | *P* value | Statistic | 95% CI | *P* value |
| FEMALE Heterosexual Sample N=105 | | | | | | | |
| Follow-up Visited/Used website  Predictors | | | | | | | |
|  | Analytical N | 105 | | | 105 | | |
|  | BA: Some College | 3.96 | 1.72, 9.13 | 0.001 | 3.06 | 1.05, 8.92 | .04 |
|  | BA: PHQ2 mean score | 1.33 | 0.95, 1.86 | 0.09 | 1.45 | 1.04, 2.00 | .03 |
| Secondary Outcomes | | | | | | | |
| Follow up PHQ2≥3  Predictors | | | | | | | |
|  | Analytical N | 105 | | | 105 | | |
|  | BA: PHQ2>=3 | 8.43 | 2.13, 33.42 | 0.002 | 11.94 | 2.46, 57.94 | .002 |
|  | BA: Total # of covid stressors | 0.83 | 0.70, 0.98 | 0.03 | 0.80 | 0.65, 0.97 | .02 |
| Follow-up GAD2≥3  Predictors | | | | | | | |
|  | Analytical N | 104 | | | 104 | | |
|  | BA: GAD2≥3 | 13.36 | 4.36, 40.95 | <.001 | 12.97 | 1.93, 42.77 | <.001 |
| Primary Outcomes  PHQ2 mean score post-pre change  Predictor | | | | | | | |
|  | Analytical N | 103 | | | 103 | | |
|  | BA: Caucasian/White | 0.59 | 0.04, 1.21 | 0.07 | 0.62 | 0.00, 1.24 | .05 |
|  | BA: Total # of Covid Stressors | -0.08 | -0.15, -0.02 | 0.01 | -0.09 | -0.16, -0.02 | .02 |
| GAD2 mean score post-pre change  Predictor | | | | | | | |
|  | Analytical N | 104 | | | 104 | | |
|  | BA: Total # of Covid stressors | -0.09 | -0.14, -0.03 | 0.002 | -0.10 | -0.16, -0.04 | .001 |
| MALE Heterosexual Sample N=43 | | | | | | | |
| Follow-up Visited/Used website  Predictors | | | | | | | |
|  | Analytical N | 43 | | | 43 | | |
|  | BA: Age in years | 0.92 | 0.87, 0.97 | 0.004 | 0.91 | 0.86, 0.96 | .001 |
| Secondary Outcomes | | | | | | | |
| Follow up PHQ2≥3  Predictors | | | | | | | |
|  | Analytical N | 43 | | |  | | |
| NO PREDICTORS ARE SIGNIFICANT | | | | | | | |
| Follow-up GAD2≥3  Predictors | | | | | | | |
|  | Analytical N | 40 | | | 40 | | |
|  | BA: GAD2≥3 | 20.66 | 2.61, 163.79 | .004 | 13.37 | 1.55, 115.02 | .02 |
| Primary Outcomes  PHQ2 mean score post-pre change  Predictor | | | | | | | |
|  | Analytical N | 39 | | | 39 | | |
|  | BA: Latinx or Spanish speaker | 0.62 | 0.94, 1.85 | .04 | 0.91 | 0.07, 1.75 | .04 |
| GAD2 mean score post-pre change  Predictor | | | | | | | |
|  | Analytical N | 38 | | | 38 | | |
|  | BA: Latinx or Spanish speaker | 0.89 | 0.17, 1.62 | .02 | 0.86 | 0.24, 1.49 | .008 |
|  | BA: Some college or less | -1.03 | -1.69, -0.36 | .003 | -0.98 | -1.67, -0.29 | .006 |
| ^a^IPW: inverse probability weighting for nonresponse predictors at baseline and follow-up.  ^b^The effect is presented as the odds ratio for dichotomous follow-up scores (e.g., PhQ≥3) and as β for mean (e.g., post-pre change in mean scores).  ^d^T4W/Juntos: Together for Wellness/Juntos por Nuestro Bienestar. | | | | | | | |
